# *In silico *microdissection of microarray data from heterogeneous cell populations

**DOI:** 10.1186/1471-2105-6-54

**Published:** 2005-03-14

**Authors:** Harri Lähdesmäki, llya Shmulevich, Valerie Dunmire, Olli Yli-Harja, Wei Zhang

**Affiliations:** 1 Institute of Signal Processing, Tampere University of Technology, P.O.Box 553, 33101 Tampere, Finland; 2 Cancer Genomics Laboratory, University of Texas M. D. Anderson Cancer Center, 1515 Holcombe Blvd., Box 85, Houston, TX 77030, USA

## Abstract

**Background:**

Very few analytical approaches have been reported to resolve the variability in microarray measurements stemming from sample heterogeneity. For example, tissue samples used in cancer studies are usually contaminated with the surrounding or infiltrating cell types. This heterogeneity in the sample preparation hinders further statistical analysis, significantly so if different samples contain different proportions of these cell types. Thus, sample heterogeneity can result in the identification of differentially expressed genes that may be unrelated to the biological question being studied. Similarly, irrelevant gene combinations can be discovered in the case of gene expression based classification.

**Results:**

We propose a computational framework for removing the effects of sample heterogeneity by "microdissecting" microarray data *in silico*. The computational method provides estimates of the expression values of the pure (non-heterogeneous) cell samples. The inversion of the sample heterogeneity can be facilitated by providing accurate estimates of the mixing percentages of different cell types in each measurement. For those cases where no such information is available, we develop an optimization-based method for joint estimation of the mixing percentages and the expression values of the pure cell samples. We also consider the problem of selecting the correct number of cell types.

**Conclusion:**

The efficiency of the proposed methods is illustrated by applying them to a carefully controlled cDNA microarray data obtained from heterogeneous samples. The results demonstrate that the methods are capable of reconstructing both the sample and cell type specific expression values from heterogeneous mixtures and that the mixing percentages of different cell types can also be estimated. Furthermore, a general purpose model selection method can be used to select the correct number of cell types.

## Background

Recent developments in high-throughput genomic technologies have revolutionized the approaches aimed at understanding biological systems and emphasized the need for computational and systems biology research. Microarray analysis, for instance, can provide massive amounts of information about a biological sample by simultaneously measuring thousands of transcript levels. Application of such methodologies has already yielded important molecular insight into cellular phenotypes under various experimental conditions [1] and provided new knowledge about the development and treatment of human diseases, such as cancers [2-4]. During the last several years, microarray technology has undergone continued improvement with better quality control in the overall measurement process, ranging from hybridization conditions to image processing techniques [5]. Nevertheless, to fully harness the power of the microarray technology to study biological materials such as cancer tissues, one has to deal with a source of measurement variability that comes from the biological materials themselves, which rarely consist of homogeneous cell populations. For example, except for a few types of immune-privileged tissues such as the brain, most solid tumor tissues contain infiltrating lymphocytes as a result of the immune response. Most tumor tissues also contain endothelial cells as part of the necessary vasculature systems that provide nutrients for the tumor cells. The complexity of this problem is that different tumor tissues contain different proportions of these non-tumor cells. Therefore, if tumor tissues are used without consideration of such a mixing phenomenon, measurement of differential gene expression will certainly be confounded by the heterogeneous cell populations. In some studies [6], pathologists carefully evaluated the tissues and only selected tissues with more than a certain percentage of tumor cells. This prescreening step, however, results in the exclusion of many tumor tissues for the study and contributes to the small sample size problem in some of the studies. Alternatively, laser capture microdissection (LCM) technology can be used to purify the tumor cells from mixed populations [7]. This approach has been very successful in DNA-based studies because of the relatively high stability of DNA. However, for microarray studies, which require less stable RNA, LCM has seen limited success because it is much more challenging to maintain RNA stability during the microdissection process. Other drawbacks of LCM are that such procedures are time-consuming and yield insufficient quantities of RNA, thus requiring multiple amplification steps that may confound quantitative inferences from gene expression data.

A recent paper by Ghosh [8] introduced a mixture model based framework for determining differential expression in the presence of mixed cell populations. In this study, we aim at reconstructing the actual expression values of the pure cell types from the heterogeneous mixtures. That is, we develop a computational method for removing the effect of mixing from heterogeneous samples and to microdissect microarray data *in silico*. Similar analytical approaches have been previously proposed by Lu *et al*. [9], Stuart *et al*. [10] and Venet *et al*. [11]. Lu *et al*. focused on estimating the fraction of cells in different phases of the cell cycle whereas Stuart *et al*. considered the problem of estimating the cell type specific expression patterns over all samples. Here we focus on estimating both the sample and cell type specific expression values using carefully controlled microarray experiments. The inversion of the 'cell mixing effect' can be made appreciably easier by providing estimates of the mixing percentages of different cell types in each measurement, which can be measured by an experienced pathologist. The entire process does not hinge upon such measurements, however, as the mixing percentages can be estimated within the modeling framework. Venet *et al*. [11] introduced some preliminary methods and results for tackling the same problem as we consider here. In particular, they used a similar regression based framework as in [10] and as we do. We also consider the problem of selecting the correct number of cell types using the cross-validation model selection framework.

## Results

The microarray data to which we apply our computational methods consists of five different heterogeneous mixtures of lymph node and colon cancer samples which are hereafter abbreviated as normal and RKO, respectively. For more details, see Materials and methods Section. Each heterogeneous mixture consists of different fractions of different cell samples, see Table 3.

### Inversion of sample heterogeneity

The first goal is to invert the mixing effect caused by sample heterogeneity. We apply the linear model developed in Materials and methods Section to the heterogeneous microarray data. The obtained results are presented below.

**Figure 1:**
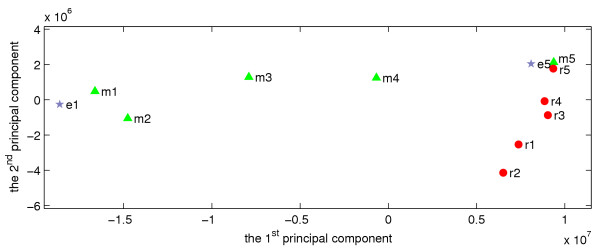
**Results of the sample heterogeneity inversion in the 2-dimensional PCA space**. All five heterogeneous samples are used to estimate the expression profiles of the pure colon cancer cells and lymphocytes. Symbols: estimated expression profiles of the pure colon cancer cells and lymphocytes (gray stars), mixture samples (green triangles), and reference samples (red circles). The labels next to each green triangle (resp. red circle) denote the number of the heterogeneous (resp. reference) sample, e.g., 'm1' = mixture sample #1 and 'r1' = reference sample #1, etc. (see also Table 3). The estimated expression profile of the pure colon cancer cells and lymphocytes have labels 'e1' and 'e5', respectively. See text for further details.

**Figure 2:**
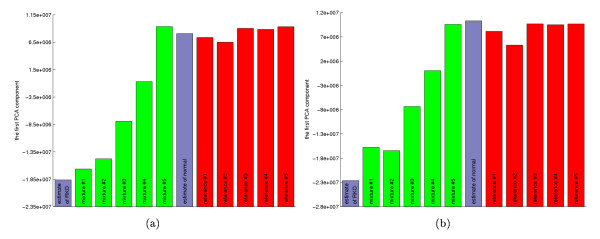
**Results of the sample heterogeneity inversion in the 1-dimensional PCA space**. (a) All five heterogeneous samples, and (b) only the heterogeneous samples #2, #3, and #4 are used to estimate the expression profiles of the pure colon cancer cells and lymphocytes. The height of each bar corresponds to the value of the most significant PCA component. Each bar corresponds to a heterogeneous sample, reference sample, or estimated expression profile and is labelled with the corresponding text.

Because of the inherent variability of individual gene expression values, the performance of the inversion method cannot be estimated based on results for individual genes. (For illustration purposes, the results of inversion of the mixing effect for individual genes are discussed and shown later on in connection with Figures 6 and 7.) Thus, we examine the performance of our method globally, by comparing the measured and estimated expression values of all the genes simultaneously. For performance evaluation and visualization purposes, the dimensionality of the 4704-dimensional expression profiles is reduced using the standard principal component analysis (PCA). The effect of the sample heterogeneity is the same for all the genes within one array. Therefore, for each array, it is useful to combine the results over all the genes. In other words, instead of looking at individual genes, we combine the expression values of all the genes and visualize the results using the most significant principal components. For comparison purposes, we also show the samples used as a reference in the conducted microarray experiments. Since the number of measurements is far smaller than the number of genes, we use a standard approach when solving the PCA eigenvector-eigenvalue problem. Let  and , *i *= 1, ..., *K*, denote the measured mixture and reference samples, respectively, and let  and  denote the estimated RKO and normal expression profiles. Let , where . Instead of finding the eigenvalues of the original sample covariance matrix *D*^*T*^*D*, we compute them for the matrix *DD*^*T*^. The eigenvalues of *D*^*T*^*D *and *DD*^*T *^are the same and the eigenvectors of *D*^*T*^*D *can be obtained from the eigenvectors of *DD*^*T *^by multiplying them by *D*^*T*^. Results of the inversion of the sample heterogeneity are shown in Figures 1 and 2. In Figure 1, all five heterogeneous samples are used to estimate the expression values of the pure colon cancer and lymphocyte samples. The two most significant PCA components of all the heterogeneous samples, reference samples, and the estimated expression profile of the pure colon cancer cells and lymphocytes are shown. Figure 1 clearly shows that the heterogeneous samples ('m1' through 'm5') are located almost on a straight line in the 2-dimensional PCA space. Furthermore, the line on which the heterogeneous samples are lying is parallel to the first principal component, suggesting that the most significant variation in the data is due to the linear mixing effect. The estimated expression profile of the pure colon cancer cells and lymphocytes are close to samples number #1 and #5, respectively, indicating that the inversion of the mixing phenomenon produces reasonable results.

The results are more easily appreciated when only the most significant PCA component is shown. As discussed above, the variation in the most significant PCA component is due to the mixing effect. The results in Figure 2 (a) are as in Figure 1, but now shown in 1-dimension in order to facilitate the interpretation. Results in Figure 2 (b), in turn, are as in Figure 2 (a) except that the inversion was done using only the samples #2, #3, and #4. This represents a more difficult and realistic case, since fewer mixtures are available.

When comparing Figure 2 (a) with Figure 2 (b), one can conclude that the method performs slightly better when more samples are used to estimate the true expression profiles – a result that was expected. Overall performance, however, is good in both cases. The estimated expression values for the pure colon cancer (RKO) are close to the mixture #1, as it should be since the mixture #1 corresponds to a measurement of the pure colon cancer. Similarly, the estimated expression values of the pure lymphocytes are close to the mixture #5 as well as to all of the reference samples (note that samples used in the reference channel (Cy5) are the same lymphocytes as the ones used in the mixtures). In Figure 1 and 2 (a), the most significant PCA component and the two most significant PCA components explain about 70.0% and 81.9% of the total variation in the data, respectively. For the reduced data, for which the results are shown in Figure 2 (b), a slightly smaller fraction of the variance is explained, namely about 67.3% and 81.2%. The results obviously depend on the optimality criterion for which we used the standard least squares. Less outlier sensitive results can be obtained with robust regression methods, such as the Huber estimator with the iteratively reweighted least squares implementation [12,13] or median based regression methods [12,14]. The robust methods provided similar global results, but improved results for some individual genes that contained one or more outliers.

### Optimization of mixing percentages

**Figure 3:**
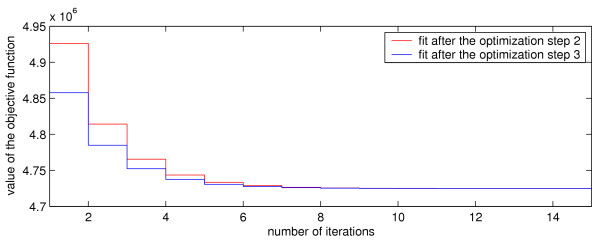
**Evolution of the value of the objective function**. The red (resp. blue) graph corresponds to the value of the objective function after step 2 (resp. step 3).

In practice, the true mixing percentages are not known but must be measured by some means. Therefore, they are also likely to contain some error. So, in addition to inverting the mixing effect, it is also useful to simultaneously estimate the most likely value of the mixing percentages. This problem can be formulated as type of optimization problem, the details of which are shown in the Materials and methods Section. The proposed optimization scheme was applied to the heterogeneous microarray data. Since the heterogeneous samples #1 and #5 correspond to the cases where only colon cancer cells and lymph node cells are used, respectively, we may assume that *α*_1 _= 1 and *α*_5 _= 0. Thus, we only estimate the value of the three remaining mixing parameters. However, practically the same results are obtained when all the five mixing parameters are estimated. We found that the convergence of the above method is practically independent of the initialization in step 1. The convergence of the optimization method is illustrated in Figure 3 by showing the evolution of the value of the objective function. Parameters in *Â*^(1) ^are initialized using the measured values shown in Table 3.

The found optimal values of the mixing percentages are shown in Table 1. The values of the estimated mixing parameters are in a good agreement with the results shown in Figure 2. That is, for instance, the heterogeneous sample #2 is quite close to the heterogeneous sample #1 (*α*_2 _≈ 0.9296) and the heterogeneous sample #4 is fairly far away from the heterogeneous sample #5 (*α*_4 _≈ 0.3796). Note that estimation of the mixing parameters may also compensate for some other errors/biases in the data than just the mixing percentages.

**Figure 4:**
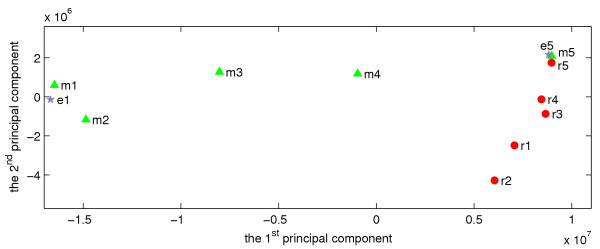
**Results of the combined sample heterogeneity inversion and the estimation of the most likely values of the mixing parameters in the 2-dimensional PCA space**. All five heterogeneous samples are used to estimate the expression profiles of the pure colon cancer and lymphocyte. Symbols: estimated expression profiles (gray stars), mixture samples (green triangles), and reference samples (red circles). See text for further details.

**Figure 5:**
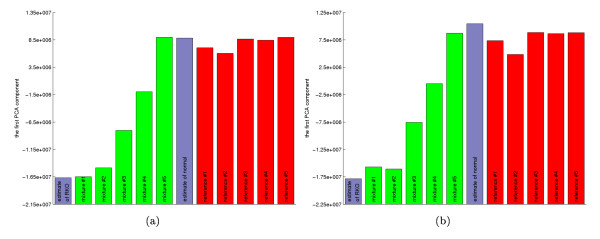
**Results of the combined sample heterogeneity inversion and the estimation of the most likely values of the mixing parameters in the 1-dimensional PCA space**. (a) All five heterogeneous samples, and (b) only the heterogeneous samples #2, #3, and #4 are used to estimate the expression profiles of the pure colon cancer cells and lymphocytes. Each bar corresponds to a heterogeneous sample, reference sample, or estimated expression profile and is labelled with the corresponding text. The height of each bar corresponds to the value of the most significant PCA component.

The obtained expression estimates for the pure colon cancer and lymph node samples, when all five heterogeneous samples are used in estimation, are shown in Figure 4. Again, the two most significant PCA components of all the heterogeneous samples, reference samples, and the estimated expression profiles of the pure colon cancer cells and lymphocytes are shown. It is instructive to compare these results with the ones shown in Figure 1. Because the heterogeneous samples are again located almost on a straight line, we use 1-dimensional visualization for the results. Figure 5 shows the obtained expression estimates in 1-dimensional PCA space.

Again, the estimated expression values for the pure colon cancer cells (RKO) are close to those from mixture #1, as it should be, since mixture *#*1 corresponds to a measurement of the pure colon cancer cells. Similarly, the estimated values from the lymph node sample are close to those from mixture #5 as well as to all of the reference samples. In Figures 4 and 5 (a), the first PCA component and the first two PCA components explain about 69.2% and 81.7% of the total variation in the data. For the reduced data, for which the results are shown in Figure 5 (b), the fractions of variance explained are about 65.1% and 80.4%. Although the fraction of variance explained is slightly smaller than without the optimization of the mixing parameters, the optimized mixing parameters provide a better fit to the data.

### Confidence intervals

Above we were only interested in estimating the expression values of the pure cell types. Often it is also useful to assess the confidence intervals of the obtained expression estimates. For that purpose, we consider two methods: one based on Gaussian approximation and the other using bootstrap. (For more details, see Materials and methods Section.)

**Figure 6:**
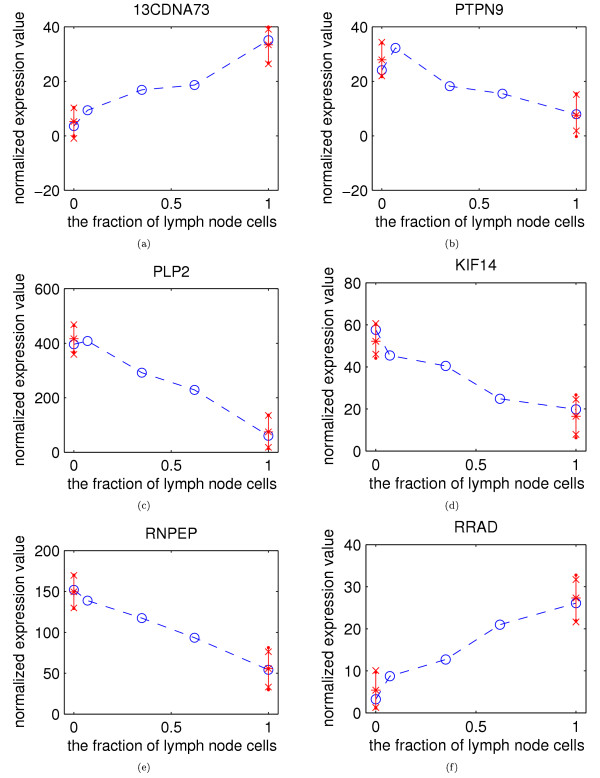
**Estimated 90 % confidence intervals for the estimated expression values of the pure cell types**. The horizontal and vertical axes correspond to the fraction of lymph node cells and the normalized expression value, respectively. Symbols: the measured expression values (blue circles), the estimated expression values of the pure cell types (red stars), regression-based confidence intervals (red points), and bootstrap-based confidence intervals (red x-marks).

For illustration purposes, Figure 6 shows the 90% estimated confidence intervals for a set of genes by pooling each of them with the 50 closest genes. The horizontal and vertical axes correspond to the fraction of lymph node cells and the normalized expression value, respectively. In other words, the different heterogeneous mixtures are placed on the *x*-axis according to the corresponding mixing fractions. The vertical lines at *x *= 0 and *x *= 1 expand over the maximum of the two confidence intervals. In most of the cases the two confidence intervals are in good agreement. The confidence intervals can be tightened by measuring more heterogeneous mixtures.

The proposed inversion methods for the sample heterogeneity were also tested on standard non-replicated microarray data by treating the replicated measurements for each gene as individual "genes." The obtained results were qualitatively similar with the ones shown above and only slightly more variable. In a similar fashion, we examined the effect of low quality replicates on the heterogeneity inversion. Slightly less variable results were obtained with a method [15] that detects and removes unreliable replicates prior to the averaging. A drawback of such unreliable spot detection is that, without any missing value estimation method, some of the genes will be excluded from further analysis.

### Selection of the number of cell types

It is known that the heterogeneous mixtures used in our experiments consist of only two cell types. However, in general case, heterogeneous mixtures may contain an unknown number of cell types. In those cases, it is useful to assess the validity of the model (i.e., the number of cell types) as well. As introduced in Materials and methods Section, the linear mixing model can be extended to incorporate more than just two cell types. We use a general purpose cross-validation for model selection. In particular, we apply the so-called leave-one-out cross-validation (LOOCV) and test the one, two, and three cell type models. (For more computational details, see Materials and methods Section.)

For the three cell type model, the number of samples does not permit us to optimize the mixing percentages for each cross-validation training data set separately. Therefore, within the cross-validation loop, we use fixed mixing percentages and only estimate the expression values. For the two and three cell type models we use the estimated mixing percentages shown in Tables 1 and 2, respectively. The relative LOOCV errors for the one, two, and three cell type models are 1.79, 1.00, and 2.28, respectively. The results suggest that the two cell type model is indeed the correct one.

## Discussion

This paper presents an inversion method for the effects of sample heterogeneity. The proposed method is successfully applied to a carefully controlled microarray data consisting of five different heterogeneous mixtures of lymph node and colon cancer samples. The results demonstrate that both the sample and cell type specific expression values can be reconstructed from heterogeneous mixtures. In some situations, such as cancer metastases in the lymph node, lymphocytes constitute a major cell type beside tumors. Hence, with careful sample preparation, the two cell type model can directly be applied to such cases. For unknown heterogeneous mixtures obtained from more complex cancer samples, the analysis may be a bit more difficult. For example, contaminating cells may include several cell types, such as fibroblasts, endothelial cells, macrophages and lymphocytes. As the proposed method can be applied to any cell types and to any number of cell types, the method works in principle in more complex cases as well. Requirement for the number of measurements necessary for reliable inversion, however, increases together with the number of cell types present in the sample.

We have emphasized that proper inversion of the mixing effect results in more accurate expression values of the pure cell types. While this is true, it must be noted that clinically relevant information may also be incorporated into other populations than the pure (cancer) cells. For example, the degree of lymphocyte infiltration may be clinically important and could be used to complement microarray analysis. However, for comparative microarray analysis, it is important to make comparisons between homogeneous samples so as to minimize the confounding influence of different proportions of contaminating cell types.

### Application of 'in silico microdissection' to detection of differentially expressed genes

**Figure 7:**
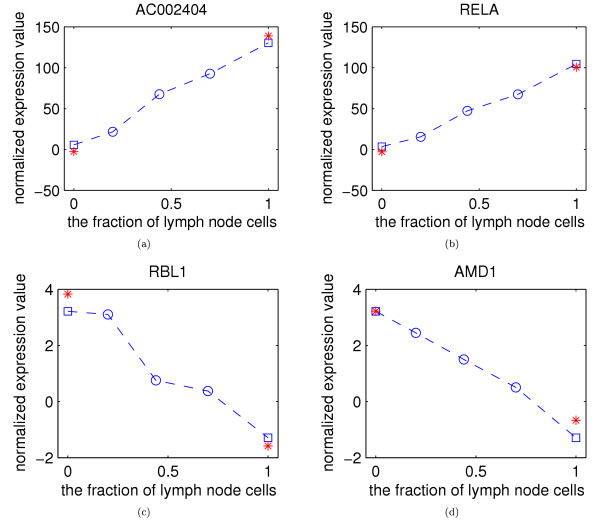
**Detecting differentially expressed genes**. A set of genes which are not found to be significantly differentially expressed based on the heterogeneous measurements (samples #2 and #4, blue circles). After the inversion of the mixing effect, however, the expression difference between the estimated pure colon cancer cells and lymphocytes (red stars) meet even a more stringent criterion of differential expression. The horizontal and vertical axes correspond to the fraction of lymph node cells and the normalized expression value, respectively. Symbols: the heterogeneous samples (blue circles), the estimated expression values (red stars), and the measured expression values of the pure colon cancer cells (blue squares). See text for more details.

In order to illustrate the above 'in silico microdissection' in practice, consider the following (hypothetical) experimental setting. Given the three middle mixture measurements (#2, #3, and #4), a goal is to identify a set of genes which are differentially expressed between the colon cancer and the lymph node samples. In a simple approach, often used in practice, the most heterogeneous sample would be discarded since it is measured to contain about 56% (resp., 44%) of colon cancer cells (resp., lymphocytes), thus giving no direct discriminative information about the underlying two samples. For illustration purposes, let us measure the expression difference of a given gene between these two samples using the fold-change, i.e., the expression value of the *i*th gene in the colon cancer sample, , is regarded as being differentially over-expressed (resp., under-expressed) if the ratio of  to the expression value of the same gene in the lymph node sample, , is at least 2 (resp., smaller than 1/2). Of course, in practice, more sophisticated methods for detecting differential expression, including correction for multiple testing, should be used. However, for illustrative purposes, this example will suffice. Since only the heterogeneous samples are available, without any inversion of the mixing effect, one must compare the mixture measurement  and . Figure 7 shows some example genes whose expression difference (i.e., the fold-change) between the two heterogeneous samples is within the given threshold (above 1/2 and below 2), but after the 'in silico microdissection,' the expression difference exceeds even a more stringent criterion (approximately 4-fold-change). The measured mixing percentages are used in the estimation (see Table 3). It is clear from this example that the proposed method is able to correctly detect differential expression even from heterogeneous samples, especially when the direct use of such samples may fail to find differential expression. Indeed, the conclusions we can draw based on the red stars are consistent with those that are based on the true homogeneous samples represented by blue squares in Figure 7.

As is evident from the example above, heterogeneity in the biological sample preparation can hinder further statistical analysis steps. Not only can the heterogeneity blur the identification of differentially expressed genes, it can also cause contrary effects. Presence of a considerable percentage of additional cell types can result in the identification of differentially expressed genes that may be unrelated to the biological question being studied. Similarly, irrelevant gene combinations can be discovered in the case of gene expression based classification. For an illustration, see [16] where the authors analyzed a colon cancer data set contaminated with muscle cells.

Although the microarray technology has been improved during the recent years, the measurements are still moderately noisy. The easiest and the most widely used approach for improving the measurement quality is to capture replicated measurements. This may become costly because each additional measurement requires an extra spot on the array, or an extra array. An alternative approach based on so-called composite microarrays was introduced in [17], where several different oligos representing different genes are printed on each spot. The multiplexing results in a mixing effect similar to the one introduced in this manuscript, and the phenomenon can be inverted to get the reconstructed expression values for single genes. The benefit is to obtain more replicated measurements without proportionately increasing the number of printed spots. Closely related ideas have also been introduced from an error-correcting microarray design point of view in [18]. The standard non-repeated microarray method does not tolerate "drop-outs": if a spot is badly corrupted and its intensity cannot be read, the expression value of the corresponding gene will be missed. Khan *et al*. showed that a certain amount of "drop-outs" can be recovered from the multiplexed samples, thus providing more error-resilient measurements. Following the methods developed in [17,18], instead of multiplexing individual genes on spots, one may wish to multiplex different samples on arrays, thus allowing a fault-tolerant recovery of expression values in the case of corrupted array(s). As a future extension, one can also consider multiplexing both the genes on spots and the samples on arrays. Similar methods for inverting the sample heterogeneity have also been studied in the context of time-series gene expression measurements in [19,20], where the fundamental mixing effect is not due to the different tissue types present in the sample, but due to the loss of synchrony of the cell population. It would be worthwhile to simultaneously study the sample heterogeneity and the loss of synchrony in the future.

## Conclusion

In this paper, we proposed a computational framework for removing the effects of sample heterogeneity. In addition to providing estimates of the expression values of the pure (non-heterogeneous) cell samples, the proposed computational methods can also be used to estimate the mixing percentages of different cell types. Furthermore, we also proposed a way of applying general-purpose model selection method for the selection of the correct number of cell types. Application of the proposed methods to a carefully controlled cDNA microarray data obtained from heterogeneous samples shows that the computational methods can invert the effect of sample heterogeneity and, at the same time, estimate the mixing percentages of the different cell types. Furthermore, a general purpose model selection method can be used to select the correct number of cell types.

## Materials and methods

### Microarray production

RNA isolation, microarray production, and microarray hybridization were carried out as described previously in [21]. RNA from normal human lymph node was purchased from a commercial source (Stratagene, La Jolla, CA). Five *μ*g aliquots of total RNA from normal lymph node and RKO colon cancer cell line were reverse transcribed using Superscript II RT (Invitrogen, Carlsbad, CA) in conjunction with oligodT-T7 primers according to the manufacturer's suggested protocol. The second strand was synthesized using 10U *E. coli *DNA ligase (vendor), 40U *E. coli *DNA polymerase I (vendor), and 2U *E. coli *Rnase H (vendor). This reaction was stopped with EDTA and then cleaned with Qiagen's PCR Purification kit (Qiagen, Valencia, CA). The double stranded cDNA was then amplified by an *in vitro *transcription reaction (Ambion, Austin, TX) and cleaned with Qiagen's Rneasy kit (Qiagen, Valencia, CA). Each amplified cRNA sample was then quantitated using a Beckman DU640 spectrophotometer (Beckman, Fullerton, CA). Five *μ*g amplified cRNA from Stratagene's normal lymph node was labeled with Cy5 for each microarray hybridization. Mixtures of appropriate volumes of cRNA from normal lymph node and RKO were labeled with Cy3 in a reverse transcription reaction using Superscript II RT (see Table 3). Labeled samples were co-hybridized overnight at 60°C in a humidified incubator on a cDNA microarray containing 4704 human genes in duplicate produced in-house. The 4704 genes represent most of the known genes in the cDNA library we used to generate the microarrays. For the purpose of this study, the identity of the genes is not very important since we only study the general effect of sample heterogeneity. As the mixing effect is the same for all the genes, we expect to have similar results when the whole genome arrays are used. Slides were scanned with an LS-IV laser scanner (Genomic Solutions, Ann Arbor, MI). In total, five different heterogeneous mixtures were measured. The measured mixing percentages are shown in Table 3.

### Preprocessing

The microarray data consists of five different heterogeneous mixtures of lymph node and colon cancer samples which are hereafter abbreviated as normal and RKO, respectively. (For more details, see Microarray production Section above and Table 3.) The gene expression data set was preprocessed as follows. The replicated background-subtracted signal intensities were averaged and log_2_-transformed, and the dye-bias effect was corrected in the log_2_-domain using the standard lowess smoothing-based normalization (see e.g. [22]) with smoothing parameter *f *= 0.7. Because the averaging effect (source of heterogeneity) takes place on the molecular level, the phenomenon must be modeled using the absolute expression values. Therefore, after the correction of the dye-bias, the data were transformed back to the original domain using the inverse of the log_2_-transformation. Correspondingly, single-channel data were used for further analysis. In order to mitigate the between array variability, the data were further standardized for each array and the two channels separately.

### Modeling sample heterogeneity

The two samples, RKO colon cancer cells and normal lymphocytes, are mixed at the extracted RNA level. Therefore, without any further verification, the model can be assumed to be linear. Lymphocytes were used because tumor tissues often contain infiltrating lymphocytes, especially in tumor metastases in the lymph nodes. Let  and  denote the expression level of the *i*th gene in the colon cancer (RKO) and in the lymph node (normal) samples, respectively. Assuming only two different cell types are mixed, the sample heterogeneity is modeled by a simple linear model



where  denotes the expression value of the *i*th gene in the *k*th heterogeneous sample, and 0 ≤ *α*_*k *_≤ *1 *denotes the fraction of the colon cancer cells in the *kth *mixture. It is worth noting that we use the same mathematical model for the sample heterogeneity as in [9-11]. Also note that in Equation (1) it is assumed that the expression level in RKO () and normal () is "fixed" and does not change between heterogeneous measurements. In other words, the measurements  come from the same heterogeneous sample with different mixing fractions. In order to allow variation in the expression values between different samples/treatments/time points, the same model can be applied separately to each set of measurements from the other samples/treatments/time points. The same model can also be extended to more than two cell types (for more details, see Selection of the number of cell types Section below).

### Inversion of sample heterogeneity

The first objective is to invert the mixing effect shown in Equation (1), that is, to obtain estimates for the expression values of the pure colon cancer cells and the pure lymphocytes. In practice, however, the measured expression values, *y*_*i*_, include one or more sources of noise. By making some distributional assumptions, one could use standard model-based estimation methods. However, in order to avoid making additional modeling assumptions, we prefer to use a general purpose least squares method to estimate the gene expression levels corresponding to the pure samples.

Let the number of genes be *n *and assume that one has measured the expression values for *K *different heterogeneous mixtures. Thus, one has measurements , 1 ≤ *i *≤ *n*, 1 ≤ *k *≤ *K*. Let us also assume for now that the mixing percentages are known or have been measured. For the *i*th gene the sample heterogeneity can be expressed as (excluding all noise terms)



When including all *n *genes, the above model can be rewritten as



where **0 **denotes the *K*-by-2 zero matrix. Let the block matrix in Equation (2) above be denoted as *Ã *Assuming the column rank of *A *is full, the well-known least squares solution is given by



where . Due to the structure of the matrix *Ã*, the least squares solution can be obtained gene-wise as .

The Gauss-Markov theorem says that the standard least squares solution is indeed the best linear unbiased estimate if the noise in the measurements is additive and i.i.d. with constant variance. However, a common observation is that the homoscedasticity does not always hold for microarray data, but instead, the noise variance depends on the underlying signal intensity [23,24]. Such heteroscedasticity may decrease the power of the inversion method shown in Equation (3). Fortunately, the structure of the matrix *Ã *ensures that the inversion can also be performed for each gene separately. Consequently, it is not necessary for the homoscedasticity to hold globally. Indeed, all we need to assume is that the noise variance is approximately constant for each gene separately.

Also note that, in this two cell type model, no prior knowledge about the expression values of either of the two cell types is needed since the method estimates the expression values for both of the two cell types. The same is also true for more general models including more cell types, assuming the model is sufficiently over-determined (see also Selection of the number of cell types Section below).

### Optimization of mixing percentages

In practice, the mixing percentages must be measured by some means. Therefore, they are also likely to contain some error. So, overall, one would like to estimate not only the expression values for the pure cell types but also the most likely value of the mixing percentages.

Assuming the model in Equation (2) is sufficiently over-determined, the mixing parameters can be adjusted computationally, too. Let us again consider the case where only two different cell types are mixed. Note that *K *denotes the number of different heterogeneous mixtures measured. Therefore, the regression matrix *Ã *in Equation (2) has only *K *free parameters. Since the number of expression values to be estimated is 2*n*, the total number of free parameters in Equation (2) is 2*n *+ *K*. The number of equations in Equation (2) is *Kn*. Hence, the model is over-determined if *Kn *> 2*n *+ *K*, which, for a fixed *n *≥ 3, holds if *K *> 2. (Note that in our case we have measured five different heterogeneous mixtures, i.e., *K *= 5.) As above, no assumptions on the noise distributions are being made and we use the least squares method. This results in the following optimization problem



A similar optimization problem was also introduced in [11].

**Figure 8:**
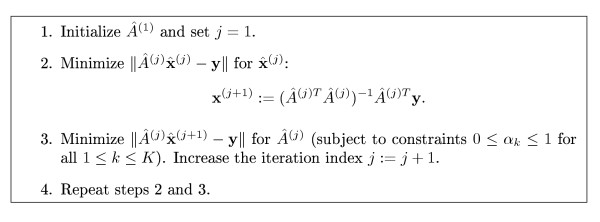
**The two-step optimization algorithm**. Details of the two-step algorithm used for the optimization problem shown in Equation (4).

Because the objective function in Equation (4) above is minimized over both *Ã *and **x**, the objective function is not linear in the parameters anymore and, therefore, cannot be solved as in Equation (3). In general, any iterative optimization method can be used to get a solution. Iterative methods usually become inefficient/unstable as the number of parameters to be optimized increases. In this case, the number of free parameters in *Ã *and **x **is 2*n *+ *K*. Therefore, we use a two-step approach in the optimization. In the first step, given proper initial value for *Ã*, the least squares solution for **x **is found using Equation (3). In the second step, the mixing percentages are optimized in the least squares sense (subject to the constraints 0 ≤ *α*_*k *_≤ 1 for all 1 ≤ *k *≤ *K*) using the previously found value for **x**. These two steps are then repeated, essentially resulting in a type of expectation-maximization (EM) approach. A similar iterative procedure was also proposed in [11], except with different constraints. Note that when Equation (4) is minimized over *Ã*, given the value of **x**, the optimization problem is again linear in its parameters. Assuming the constraints are not violated, the standard equation (similar to the one in Equation (3)) can be applied. If that is not the case, then any general-purpose constrained optimization method may be applied. Let  (resp. *Â*^(*j*)^) denote the value of **x **(resp. *Ã*) after the *j*th iteration. Details of the algorithm are shown in Figure 8. Clearly, at each iteration of steps 2 and 3, the value of the objective function is decreased. Thus, a minimum will be found.

### Confidence intervals

It is useful to assess the confidence intervals of the obtained expression estimates. As explained above, the Gauss-Markov theorem is applied gene-wise that greatly alleviates the issue of heteroscedasticity. Should the noise variance *σ*^2 ^be constant, then the variance of the estimated expression values would be . Due to the special structure of the matrix *Ã *(i.e., the gene-wise inversion of the mixing effect), the variance of the estimated expression values for the *i*th gene can be expressed as



where  is the noise variance for the *i*th gene. A straightforward way of obtaining an estimate of the variance is to compute the sample noise variance  for each gene and then apply Equation (5) to get . That would result in somewhat sensitive variance estimates since there are only *K *= 5 error residuals associated with each gene. A better alternative is to pool genes which have approximately the same average expression value  and then compute the sample noise variance from the error residuals of the pooled genes. Although we do not assume Gaussian noise distribution, we can resort to the Gaussian approximation when computing the confidence intervals. For example, using the Gaussian approximation, the 1 - 2*α *confidence interval for estimated expression value of the *i*th gene in the colon cancer cells is , where Φ^-1^(·) is the inverse of the standard normal cumulative distribution function and  denotes the (1,1) element of the estimated variance matrix  (similarly for the lymph node sample: ). Alternatively, the confidence intervals can be obtained using the non-parametric bootstrap framework [25]. Here we consider the method in which one re-samples the error residuals with replacement (within the set of pooled genes) and computes the confidence intervals directly from the *α *and 1 - *α *percentiles of the bootstrap distribution of the expression estimates.

### Selection of the number of cell types

Although it is known that only two cell types are mixed in our experiments there may be other experimental settings where the number of cell types may be unknown. Then it is useful to assess the validity of the model as well. As was mentioned above, the linear mixing model can be extended to incorporate more than just two cell types using a straightforward extension: , where  denotes the expression value of the *i*th gene in the *j*th cell type, and 0 ≤  ≤ 1 denotes the fraction of the *j*th cell type in the *k*th mixture. The mixing percentages must also satisfy  for all *k*. The significance of different 'regression coefficients'  could be tested using standard regression-based statistical tests. Since those tests apply only to Gaussian noise we recommend using a general purpose cross-validation for model selection (see e.g. [26]). Here we consider the leave-one-out cross-validation (LOOCV) and test the one, two, and three cell type models. Thus, each heterogeneous sample is left out from the training data at a time, the regression coefficients are estimated based on the remaining four samples, and the model is then tested on the sample which was left out from the training data set.

## Authors' contributions

VD and WZ conducted the experiments and HL, IS, OY-H and WZ developed the computational methods. HL, IS and WZ prepared the manuscript.

## Figures and Tables

**Table 1:** The estimated mixing percentages. The found optimal values of the mixing percentages.

	sample #2	sample #3	sample #4
RKO	92.96	65.06	37.96
normal	7.04	34.94	62.04

**Table 2:** The estimated mixing percentages for the three cell type model. The found optimal values of the mixing percentages for the three cell type model.

cell type	sample #1	sample #2	sample #3	sample #4	sample #5
RKO	98.15	67.94	58.70	30.74	0
normal	1.22	16.39	36.53	62.97	96.95
the 3rd cell type	0.63	15.66	4.77	6.29	3.05

**Table 3:** The measured mixing percentages. The measured mixing percentages (RKO/normal) in the five heterogeneous samples.

	sample #1	sample #2	sample #3	sample #4	sample #5
RKO	100	80	56	30	0
normal	0	20	44	70	100
